# Spotlight on landmark oncology trials: the latest evidence and novel trial designs

**DOI:** 10.1186/s12916-017-0884-7

**Published:** 2017-06-02

**Authors:** Helena Earl, Stefano Molica, Piotr Rutkowski

**Affiliations:** 1 University of Cambridge Department of Oncology, NIHR Cambridge Biomedical Research Centre, and Hon Consultant in Medical Oncology, Cambridge University Hospital NHS Foundation Trust, Cambridge, UK; 2 Department Hematology-Oncology, Azienda Ospedaliera Pugliese-Ciaccio, 88100 Catanzaro, Italy; 3 Department of Soft Tissue/Bone Sarcoma and Melanoma, Maria Sklodowska-Curie Institute - Oncology Center, Roentgena 5, 02-781 Warsaw, Poland

**Keywords:** Cancer, Clinical trials, Oncology, Randomised trials, Targeted therapy, Trial design

## Abstract

The era of precision oncology is marked with prominent successes in the therapy of advanced soft tissue sarcomas, breast cancer, ovarian cancer and haematological neoplasms, among others. Moreover, recent trials of immune checkpoint inhibitors in melanoma, non-small cell lung carcinoma, and head and neck cancers have significantly influenced the therapeutic landscape by providing promising evidence for immunotherapy efficacy in the adjuvant setting in high-risk locoregional disease. To speed up the introduction of targeted therapy for cancer patients, novel phase II trials are being designed, and may likely form the basis for the ‘landmark trials’ of the future. A special article collection in *BMC Medicine*, “Spotlight on landmark oncology trials”, features articles from invited experts on recent clinical practice-changing trials.

## Background

The era of precision medicine has led to significant developments in the therapy of advanced soft tissue sarcomas (STS), breast cancer, ovarian cancer and haematological neoplasms, among others. However, cancer research also faces challenges in the effective development and assessment of targeted therapeutics [1], including the need for early evaluation of potential biomarkers by translational and correlative studies.

In this editorial, we discuss the special article collection entitled “Spotlight on landmark oncology trials” recently published in *BMC Medicine*, which focuses on the core clinical trials of selected solid tumours (lung cancer [2], melanoma [3, 4], STS [5], head and neck cancer [6]). We also highlight selected and recent practice-changing trials in chronic lymphocytic leukaemia as well as breast and gynaecological cancers, and review the advances offered by the development of novel clinical trial designs.

## Recent landmark immunotherapy trials – melanoma, non-small cell lung carcinoma (NSCLC), head and neck cancer

The first articles in the special article collection focus on landmark clinical trials in selected advanced solid tumours, with special attention on the most studied tumours with regards to immunotherapy development, namely melanoma [3, 4], NSCLC [2], and head and neck cancer [6]. Recent developments and approvals in immunotherapy have significantly changed the landscape of melanoma and NSCLC therapy in the metastatic setting, and open various possibilities for adjuvant treatment in high-risk locoregional disease [7–10]. In this article series, worldwide renowned experts in their fields provided an extensive overview on the state of the art in immunotherapy and discussed the possible future paths in these, still difficult, types of malignancies.

The current results of anti-PD-1 therapy with pembrolizumab or nivolumab monotherapy in melanoma indicated a median overall survival (OS) of approximately 2 years, but the combination of anti-PD-1 and anti-CTLA-4 (nivolumab with ipilimumab) was shown to be superior in terms of progression-free survival (PFS) and OS (Table 1) [11–15]. Further clinical trials are under way to determine how best to integrate combination immunotherapy and other treatment modalities as well as to establish the correct sequence of therapy with targeted treatment in *BRAF*-mutated cases.

**Table 1:** Summary of major trials with immunotherapy in advanced cutaneous melanoma

Study	N (% 1st line)	ORR	Median PFS	2-year OS rate
Pembrolizumab (10 mg/kg every 2 weeks) KEYNOTE-006 Robert et al., 2015 [11] Schachter et al., 2016 [12]	279 (65.6%)	33.7%	5.6 months	55.0%
Pembrolizumab (10 mg/kg every 3 weeks) KEYNOTE-006 Robert et al., 2015 [11] Schachter et al., 2016 [12]	277 (66.8%)	32.9%	4.1 months	55.0%
Nivolumab in *BRAF*-negative melanoma Checkmate-066 Robert et al., 2015 [13]	210 (100%)	40.0%	5.4 months	57.7%
Ipilimumab + Nivolumab Checkmate-067 Larkin et al., 2015 [14] Larkin et al., 2017 [15]	314 (100%)	58.9%	11.7 months	64.0%

*ORR* overall response rate; *OS* overall survival; *PFS* progression-free survival

The team lead by Professor Jean-Charles Soria discussed the successes and failures of immunotherapy in the first-line treatment of NSCLC [2]. Moreover, three anti-PD-1/anti-PD-L1 agents, pembrolizumab, nivolumab and atezolizumab, have been approved for second-line therapy of NSCLC [16–18]; however, contrary to melanoma, patient selection to therapy should be based on PD-L1 expression level of tumour cells.

## Recent landmark trials in STS

Another topic featured in this article collection is systemic therapy in STS [5], which is a heterogeneous group of rare solid tumours. Despite optimal local treatment, approximately 50% of adult patients with localised STS develop distant metastases and die of metastatic disease. A limited number of drugs have shown activity in advanced disease, and due to the rarity of these tumours, clinical trials in sarcoma include many subtypes and are mainly initiated by academic research groups.

Recent developments in the classification of STS, insights into their molecular pathogenesis and the optimal treatment strategies have evolved considerably during the past decades and have led to the introduction of new therapies. Nevertheless, the selection of systemic therapy must be strictly individualised and based upon several factors, including the histology and biological behaviour of the disease. A summary of recent pivotal trials for systemic therapy in advanced STS is presented in Table 2 [19–22].

**Table 2:** Summary of recent pivotal clinical trials in advanced soft tissue sarcomas

Tumour type, phase [reference]	Line of therapy	Arms (experimental vs. control)	Response rate	Clinical benefit rate	Median PFS (months, *P* value)	Median OS (months, *P* value)
Non-adipocytic soft tissue sarcoma, phase 3, *n* = 369 [19]	Second or later (after anthracycline)	Pazopanib 800 mg/m^2^ vs. placebo	6% vs. 0%	73% vs. 38%	4.6 vs. 1.6 (*P* < 0.0001)	12.5 vs. 10.7 (*P* = 0.25)
Liposarcoma and leiomyosarcoma, phase 3, *n* = 518 [20]	Second or later (after anthracycline)	Trabectedine 1.5 mg/m^2^ vs. dacarbazine 1000 mg/m^2^	10% vs. 7%	61% vs. 42%	4.2 vs. 1.5 (*P* < 0.001)	12.4 vs. 12.9 (*P* = 0.37)
Liposarcoma and leiomyosarcoma, phase 3, *n* = 452 [21]	Third or later (after anthracycline)	Eribulin mesylate 1.4 mg/m^2^ vs. dacarbazine 850–1200 mg/m^2^	5% vs. 4%	57% vs. 52%	2.6 vs. 2.6 (*P* = 0.23)	13.5 vs. 11.5 (*P* = 0.01)
Soft tissue sarcoma, phase 2, *n* = 133 [22]	First line	Olaratumab 15 mg/kg plus doxorubicin 75 mg/m^2^ vs. doxorubicin alone 75 mg/m^2^	18.2% vs. 11.9%	77.3% vs. 62.7%	6.6 vs. 4.1 (*P* = 0.06)	26.5 vs. 14.7 (*P* = 0.0003)

*OS* overall survival; *PFS* progression-free survival

## Recent landmark trials in breast and gynaecological cancers

Recent landmark trials in HER2-positive breast cancer include those using dual HER2-targeted therapy pertuzumab and trastuzumab with docetaxel. In the neoadjuvant setting, the NeoSphere trial demonstrated significantly improved pathological complete response rates [23] and a trend favouring improved PFS and OS at 5 years [24]. Results from the CLEOPATRA trial in the metastatic setting of the same treatment have produced remarkable results [25]; the same combination produced a 56.5-month median OS compared with 40.8 months achieved with trastuzumab and docetaxel alone, showing an increase of 15.7 months to OS in the pertuzumab group. These results clearly demonstrate the superiority of dual HER2-directed therapy. In ER-positive, HER2-negative metastatic disease, the landmark trial (PALOMA 3) uses the CDK 4/6 inhibitor, palbociclib [26, 27]. Median PFS was 9.5 months in the fulvestrant plus palbociclib group and 4.6 months in the fulvestrant plus placebo group with a hazard ratio of 0.46, which was highly statistically significant. However, translational research did not discover any predictive biomarker subgroups [27] for the palbociclib effect.

The landmark phase III trials in high-grade serous ovarian cancer are testing PARP inhibitors as maintenance therapy after response to platinum-based therapy in relapsed disease. Study 19 [28, 29] used olaparib against placebo and demonstrated a PFS of 11.2 months in *BRCA*-mutated patients compared with 4.3 months for wild-type patients (hazard ratio, 0.18; *P* < 0.0001). A more recent niraparib study had similar results [30], where patients in the niraparib group had a significantly longer PFS than the placebo group in all cohorts tested (21.0 vs. 5.5 months in the g*BRCA* cohort; 12.9 vs. 3.8 months in the non-g*BRCA* cohort for patients who had tumours with homologous recombination deficiency; and 9.3 vs. 3.9 months in the overall non-g*BRCA* cohort; *P* < 0.001). Both trials demonstrated significant benefit for maintenance PARP inhibitors in all subgroups of platinum-sensitive relapsed high-grade serous ovarian cancer.

## Recent landmark trial in chronic lymphocytic leukaemia (CLL): upfront therapy with ibrutinib in elderly with chronic lymphocytic leukaemia (The RESONATE-2 Trial)

With the advent of novel oral agents that are well tolerated and highly efficacious, the therapeutic landscape of CLL underwent radical changes [31]. In phase 3 trials, ibrutinib, a first-in-class Bruton tyrosine kinase (BTK) inhibitor, showed efficacy over traditional salvage therapeutic options in patients with relapsed or refractory CLL [32]. The importance of BTK inhibitors in the first-line setting has been recently investigated in the RESONATE-2 study [33], a head-to-head clinical trial in which outcomes were shown to be superior for patients who received ibrutinib in comparison to patients treated with chlorambucil single agent. Indeed, ibrutinib demonstrated a survival advantage over chlorambucil despite the study’s crossover design. The strength of the study also relies on the good tolerance profile of ibrutinib, which allows it to be administered continuously and provide indefinite disease suppression even in elderly or unfit CLL patients. However, a potential setback is represented by the control arm since chlorambucil is no longer regarded an adequate therapy in CLL [26]. Combinations of chlorambucil with an anti-CD2O monoclonal antibody, such as rituximab, ofatumumab or obinutuzumab, are now the standard of care in patients unsuited to receive fludarabine, cyclophosphamide and rituximab [34–36]. Further, a trial comparing ibrutinib plus obinutuzumab versus chlorambucil plus obinutuzumab finished recruiting, but results are not yet mature (ClinicalTrials.gov Identifier: NCT02264574). Therefore, in absence of data from this and similar trials, either therapeutic choice is adequate in the day-to-day practice.

In conclusion, the RESONATE-2 trial demonstrates that ibrutinib is a new important player in the treatment of elderly unfit patients and in those with high-risk disease. A potential shortcoming with the upfront use of ibrutinib includes cost and indefinite treatment course [37]. Finally, biomarker and minimal residual disease assessment may ultimately be useful to guide the targeted agent or regimen of choice and the duration of treatment [38].

## Novel trial designs and conclusions

The landmark oncology trials highlighted in the *BMC Medicine* series “Spotlight on landmark oncology trials” and this editorial are recent trials that have produced practice-changing results for patients. These trials represent the end of the long process of translating scientific innovation and drug discovery, through first-in-man studies, followed by phase II trials and finally by randomised phase III trials as required for licensing of new treatments. Novel trial designs could potentially lead to a different type of landmark trial that would accelerate the process and allow cancer patients to access new treatments faster. In the phase I setting, there is a pressing need to develop better trial methodologies for novel combinations, often of a standard chemotherapy with a novel targeted agent. Di Veroli et al. [39] published an interesting software to provide information in terms of synergy and/or antagonism between two compounds. In addition, adaptive designs for phase I combinations are being developed [40].

In the era of precision cancer medicine, innovative trial designs will also require the matching of novel drugs with putative targets. Indeed, BATTLE, a landmark phase II trial using an adaptive randomised design, tested four novel drugs and biomarker pairings in NSCLC [41]. This was followed by BATTLE-2 [42], testing combination treatments in the same disease. In neoadjuvant breast cancer, the I-SPY 1 and 2 trials have successfully matched treatment and biomarkers, using adaptive randomised designs [43, 44]. Landmark results include those in triple negative breast cancer for the combination of velaparib and carboplatin [44] and neratinib in HER2-positive breast cancer [45]. The FOCUS 4 trial in metastatic colorectal cancer uses group-sequential multi-arm, multi-stage methodology [46] to achieve similar matching of novel therapy and biomarker groups. Our own group is developing a novel Bayesian, adaptive randomised methodology [47]. These designs would allow recruitment of biomarker-negative patients, often not included in other trials, and have the potential for ‘perpetual’ designs, in which successful matching of novel drugs and biomarkers would result in ‘graduation’ of the pair to a phase III trial, along with the rapid rejection of novel drugs that did not work. More effective and cost-efficient phase II trial designs would rapidly lead to landmark trials and practice-changing results.

## References

[1] Saad ED, Paoletti X, Burzykowski T, Buyse M (2014). Precision medicine needs randomized clinical trials. Nat Rev Clin Oncol.

[2] Remon J, Besse B, Soria JC (2017). Successes and failures: what did we learn from recent first-line treatment immunotherapy trials in non-small cell lung cancer?. BMC Med.

[3] Rutkowski P, Kozak K (2017). News from the melanoma sessions of the European Cancer Congress 2017. BMC Med.

[4] Redman JM, Gibney GT, Atkins MB (2016). Advances in immunotherapy for melanoma. BMC Med.

[5] Frezza AM, Stacchiotti S, Gronchi A (2017). Systemic treatment in advanced soft tissue sarcoma: what is standard, what is new. BMC Med.

[6] Szturz P, Vermorken JB (2017). Immunotherapy in head and neck cancer: aiming at EXTREME precision. BMC Med.

[7] Ugurel S, Roehmel J, Ascierto PA, Flaherty KT, Grob JJ, Hauschild A, Larkin J, Long GV, Lorigan P, McArthur GA, Ribas A, Robert C, Schadendorf D, Garbe C (2016). Survival of patient s with advanced metastatic melanoma: the impact of novel therapies. Eur J Cancer.

[8] Coit DG, Thompson JA, Algazi A, Andtbacka R, Bichakjian CK, Carson WE, Daniels GA, DiMaio D, Ernstoff M, Fields RC, Fleming MD, Gonzalez R, Guild V, Halpern AC, Hodi FS, Joseph RW, Lange JR, Martini MC, Materin MA, Olszanski AJ, Ross MI, Salama AK, Skitzki J, Sosman J, Swetter SM, Tanabe KK, Torres-Roca JF, Trisal V, Urist MM, McMillian N, Melanoma EA (2016). Version 2.2016, NCCN Clinical Practice Guidelines in Oncology. J Natl Compr Canc Netw.

[9] Ettinger DS, Wood DE, Aisner DL, Akerley W, Bauman J, Chirieac LR, D’Amico TA, DeCamp MM, Dilling TJ, Dobelbower M, Doebele RC, Govindan R, Gubens MA, Hennon M, Horn L, Komaki R, Lackner RP, Lanuti M, Leal TA, Leisch LJ, Lilenbaum R, Lin J, Loo BW, Martins R, Otterson GA, Reckamp K, Riely GJ, Schild SE, Shapiro TA, Stevenson J, Swanson SJ, Tauer K, Yang SC, Gregory K, Hughes M (2017). Non-Small Cell Lung Cancer, Version 5.2017, NCCN Clinical Practice Guidelines in Oncology. J Natl Compr Canc Netw.

[10] Eggermont AM, Chiarion-Sileni V, Grob JJ, Dummer R, Wolchok JD, Schmidt H, Hamid O, Robert C, Ascierto PA, Richards JM, Lebbé C, Ferraresi V, Smylie M, Weber JS, Maio M, Bastholt L, Mortier L, Thomas L, Tahir S, Hauschild A, Hassel JC, Hodi FS, Taitt C, de Pril V, de Schaetzen G, Suciu S, Testori A (2016). Prolonged survival in stage III melanoma with ipilimumab adjuvant therapy. N Engl J Med.

[11] Robert C, Schachter J, Long GV, Arance A, Grob JJ, Mortier L, Daud A, Carlino MS, McNeil C, Lotem M, Larkin J, Lorigan P, Neyns B, Blank CU, Hamid O, Mateus C, Shapira-Frommer R, Kosh M, Zhou H, Ibrahim N, Ebbinghaus S, Ribas A (2015). KEYNOTE-006 investigators. Pembrolizumab versus ipilimumab in advanced melanoma. N Engl J Med.

[12] Schachter J, Ribas A, Long GV, Arance A, Grob JJ, Mortier L, Daud A, Carlino MS, McNeil CM, Lotem M, Larkin JMG, Lorigan P, Neyns B, Blank CU, Petrella TM, Hamid O, Zhou H, Ebbinghaus S, Ibrahim N, Robert C. Pembrolizumab versus ipilimumab for advanced melanoma: Final overall survival analysis of KEYNOTE-006. J Clin Oncol. 2016;34(Suppl; abstr 9504).

[13] Robert C, Long GV, Brady B, Dutriaux C, Maio M, Mortier L, Hassel JC, Rutkowski P, McNeil C, Kalinka-Warzocha E, Savage KJ, Hernberg MM, Lebbé C, Charles J, Mihalcioiu C, Chiarion-Sileni V, Mauch C, Cognetti F, Arance A, Schmidt H, Schadendorf D, Gogas H, Lundgren-Eriksson L, Horak C, Sharkey B, Waxman IM, Atkinson V, Ascierto PA (2015). Nivolumab in previously untreated melanoma without BRAF mutation. N Engl J Med.

[14] Larkin J, Chiarion-Sileni V, Gonzalez R, Grob JJ, Cowey CL, Lao CD, Schadendorf D, Dummer R, Smylie M, Rutkowski P, Ferrucci PF, Hill A, Wagstaff J, Carlino MS, Haanen JB, Maio M, Marquez-Rodas I, McArthur GA, Ascierto PA, Long GV, Callahan MK, Postow MA, Grossmann K, Sznol M, Dreno B, Bastholt L, Yang A, Rollin LM, Horak C, Hodi FS, Wolchok JD (2015). Combined nivolumab and ipilimumab or monotherapy in untreated melanoma. N Engl J Med.

[15] Larkin J, Chiarion-Sileni V, Gonzalez R, et al. Overall survival results from a phase III trial of nivolumab combined with ipilimumab in treatment-naïve patients with advanced melanoma (CheckMate 067). In: Proceedings from the American Association for Cancer Research Annual Meeting, April 2–5, 2017, Washington DC. Abstract CT075.

[16] Borghaei H, Paz-Ares L, Horn L, Spigel DR, Steins M, Ready NE, Chow LQ, Vokes EE, Felip E, Holgado E, Barlesi F, Kohlhäufl M, Arrieta O, Burgio MA, Fayette J, Lena H, Poddubskaya E, Gerber DE, Gettinger SN, Rudin CM, Rizvi N, Crinò L, Blumenschein GR, Antonia SJ, Dorange C, Harbison CT, Graf Finckenstein F, Brahmer JR (2015). Nivolumab versus docetaxel in advanced nonsquamous non-small-cell lung cancer. N Engl J Med.

[17] Herbst RS, Baas P, Kim DW, Felip E, Pérez-Gracia JL, Han JY, Molina J, Kim JH, Arvis CD, Ahn MJ, Majem M, Fidler MJ, de Castro G, Garrido M, Lubiniecki GM, Shentu Y, Im E, Dolled-Filhart M, Garon EB (2016). Pembrolizumab versus docetaxel for previously treated, PD-L1-positive, advanced non-small-cell lung cancer (KEYNOTE-010): a randomized controlled trial. Lancet.

[18] Fehrenbacher L, Spira A, Ballinger M, Kowanetz M, Vansteenkiste J, Mazieres J, Park K, Smith D, Artal-Cortes A, Lewanski C, Braiteh F, Waterkamp D, He P, Zou W, Chen DS, Yi J, Sandler A, Rittmeyer A, POPLAR Study Group (2016). Atezolizumab versus docetaxel for patients with previously treated nonsmall-cell lung cancer (POPLAR): a multicentre, open label, phase 2 randomised controlled trial. Lancet.

[19] van der Graaf WT, Blay JY, Chawla SP, Kim DW, Bui-Nguyen B, Casali PG, Schöffski P, Aglietta M, Staddon AP, Beppu Y, Le Cesne A, Gelderblom H, Judson IR, Araki N, Ouali M, Marreaud S, Hodge R, Dewji MR, Coens C, Demetri GD, Fletcher CD, Dei Tos AP, Hohenberger P, EORTC Soft Tissue and Bone Sarcoma Group; PALETTE study group (2012). Pazopanib for metastatic soft-tissue sarcoma (PALETTE): a randomised, double-blind, placebo-controlled phase 3 trial. Lancet.

[20] Demetri GD, von Mehren M, Jones RL, Hensley ML, Schuetze SM, Staddon A, Milhem M, Elias A, Ganjoo K, Tawbi H, Van Tine BA, Spira A, Dean A, Khokhar NZ, Park YC, Knoblauch RE, Parekh TV, Maki RG, Patel SR (2016). Efficacy and safety of trabectedin or dacarbazine for metastatic liposarcoma or leiomyosarcoma after failure of conventional chemotherapy: results of a phase III randomized multicenter clinical trial. J Clin Oncol.

[21] Schöffski P, Chawla S, Maki RG, Italiano A, Gelderblom H, Choy E, Grignani G, Camargo V, Bauer S, Rha SY, Blay JY, Hohenberger P, D’Adamo D, Guo M, Chmielowski B, Le Cesne A, Demetri GD, Patel SR (2016). Eribulin versus dacarbazine in previously treated patients with advanced liposarcoma or leiomyosarcoma: a randomised, open-label, multicentre, phase 3 trial. Lancet.

[22] Tap WD, Jones RL, Van Tine BA, Chmielowski B, Elias AD, Adkins D, Agulnik M, Cooney MM, Livingston MB, Pennock G, Hameed MR, Shah GD, Qin A, Shahir A, Cronier DM, Ilaria R, Conti I, Cosaert J, Schwartz GK (2016). Olaratumab and doxorubicin versus doxorubicin alone for treatment of soft-tissue sarcoma: an open-label phase 1b and randomised phase 2 trial. Lancet.

[23] Gianni L, Pienkowski T, Im YH, Roman L, Tseng LM, Liu MC, Lluch A, Staroslawska E, de la Haba-Rodriguez J, Im SA, Pedrini JL, Poirier B, Morandi P, Semiglazov V, Srimuninnimit V, Bianchi G, Szado T, Ratnayake J, Ross G, Valagussa P (2012). Efficacy and safety of neoadjuvant pertuzumab and trastuzumab in women with locally advanced, inflammatory, or early HER2-positive breast cancer (NeoSphere): a randomised multicentre, open-label, phase 2 trial. Lancet Oncol.

[24] Gianni L, Pienkowski T, Im YH, Tseng LM, Liu MC, Lluch A, Starosławska E, de la Haba-Rodriguez J, Im SA, Pedrini JL, Poirier B, Morandi P, Semiglazov V, Srimuninnimit V, Bianchi GV, Magazzù D, McNally V, Douthwaite H, Ross G, Valagussa P (2016). 5-year analysis of neoadjuvant pertuzumab and trastuzumab in patients with locally advanced, inflammatory, or early-stage HER2-positive breast cancer (NeoSphere): a multicentre, open-label, phase 2 randomised trial. Lancet Oncol.

[25] Swain SM, Baselga J, Kim SB, Ro J, Semiglazov V, Campone M, Ciruelos E, Ferrero JM, Schneeweiss A, Heeson S, Clark E, Ross G, Benyunes MC, Cortés J, CLEOPATRA Study Group (2015). Pertuzumab, trastuzumab, and docetaxel in HER2-positive metastatic breast cancer. N Engl J Med.

[26] Turner NC, Ro J, André F, Loi S, Verma S, Iwata H, Harbeck N, Loibl S, Huang Bartlett C, Zhang K, Giorgetti C, Randolph S, Koehler M, Cristofanilli M, PALOMA3 Study Group (2015). Palbociclib in hormone-receptor-positive advanced breast cancer. N Engl J Med.

[27] Cristofanilli M, Turner NC, Bondarenko I, Ro J, Im SA, Masuda N, Colleoni M, DeMichele A, Loi S, Verma S, Iwata H, Harbeck N, Zhang K, Theall KP, Jiang Y, Bartlett CH, Koehler M, Slamon D (2016). Fulvestrant plus palbociclib versus fulvestrant plus placebo for treatment of hormone-receptor-positive, HER2-negative metastatic breast cancer that progressed on previous endocrine therapy (PALOMA-3): final analysis of the multicentre, double-blind, phase 3 randomised controlled trial. Lancet Oncol.

[28] Ledermann J, Harter P, Gourley C, Friedlander M, Vergote I, Rustin G, Scott C, Meier W, Shapira-Frommer R, Safra T, Matei D, Macpherson E, Watkins C, Carmichael J, Matulonis U (2012). Olaparib maintenance therapy in platinum-sensitive relapsed ovarian cancer. N Engl J Med.

[29] Ledermann J, Harter P, Gourley C, Friedlander M, Vergote I, Rustin G, Scott CL, Meier W, Shapira-Frommer R, Safra T, Matei D, Fielding A, Spencer S, Dougherty B, Orr M, Hodgson D, Barrett JC, Matulonis U (2014). Olaparib maintenance therapy in patients with platinum-sensitive relapsed serous ovarian cancer: a preplanned retrospective analysis of outcomes by BRCA status in a randomised phase 2 trial. Lancet Oncol.

[30] Mirza MR, Monk BJ, Herrstedt J, Oza AM, Mahner S, Redondo A, Fabbro M, Ledermann JA, Lorusso D, Vergote I, Ben-Baruch NE, Marth C, Mądry R, Christensen RD, Berek JS, Dørum A, Tinker AV, du Bois A, González-Martín A, Follana P, Benigno B, Rosenberg P, Gilbert L, Rimel BJ, Buscema J, Balser JP, Agarwal S, Matulonis UA, ENGOT-OV16/NOVA Investigators (2016). Niraparib maintenance therapy in platinum-sensitive, recurrent ovarian cancer. N Engl J Med.

[31] Molica S (2017). Targeted therapy in the treatment of chronic lymphocytic leukemia: facts, shortcomings and hopes for the future. Expert Rev Hematol.

[32] Byrd JC, Brown JR, O’Brien S, Barrientos JC, Kay NE, Reddy NM, Coutre S, Tam CS, Mulligan SP, Jaeger U, Devereux S, Barr PM, Furman RR, Kipps TJ, Cymbalista F, Pocock C, Thornton P, Caligaris-Cappio F, Robak T, Delgado J, Schuster SJ, Montillo M, Schuh A, de Vos S, Gill D, Bloor A, Dearden C, Moreno C, Jones JJ, Chu AD, Fardis M, McGreivy J, Clow F, James DF, Hillmen P, RESONATE Investigators (2014). Ibrutinib versus ofatumumab in previously treated chronic lymphoid leukemia. N Engl J Med.

[33] Burger JA, Tedeschi A, Barr PM, Robak T, Owen C, Ghia P, Bairey O, Hillmen P, Bartlett NL, Li J, Simpson D, Grosicki S, Devereux S, McCarthy H, Coutre S, Quach H, Gaidano G, Maslyak Z, Stevens DA, Janssens A, Offner F, Mayer J, O’Dwyer M, Hellmann A, Schuh A, Siddiqi T, Polliack A, Tam CS, Suri D, Cheng M, Clow F, Styles L, James DF, Kipps TJ, RESONATE-2 Investigators (2015). Ibrutinib as initial therapy for patients with chronic lymphocytic leukemia. N Engl J Med.

[34] Hillmen P, Gribben JG, Follows GA, Milligan D, Sayala HA, Moreton P, Oscier DG, Dearden CE, Kennedy DB, Pettitt AR, Nathwani A, Varghese A, Cohen D, Rawstron A, Oertel S, Pocock CF (2014). Rituximab plus chlorambucil as first-line treatment for chronic lymphocytic leukemia: Final analysis of an open-label phase II study. J Clin Oncol.

[35] Goede V, Fischer K, Busch R, Engelke A, Eichhorst B, Wendtner CM, Chagorova T, de la Serna J, Dilhuydy MS, Illmer T, Opat S, Owen CJ, Samoylova O, Kreuzer KA, Stilgenbauer S, Döhner H, Langerak AW, Ritgen M, Kneba M, Asikanius E, Humphrey K, Wenger M, Hallek M (2014). Obinutuzumab plus chlorambucil in patients with CLL and coexisting conditions. N Engl J Med.

[36] Hillmen P, Robak T, Janssens A, Babu KG, Kloczko J, Grosicki S, Doubek M, Panagiotidis P, Kimby E, Schuh A, Pettitt AR, Boyd T, Montillo M, Gupta IV, Wright O, Dixon I, Carey JL, Chang CN, Lisby S, McKeown A, Offner F, COMPLEMENT 1 Study Investigators (2015). Chlorambucil plus ofatumumab versus chlorambucil alone in previously untreated patients with chronic lymphocytic leukaemia (COMPLEMENT 1): a randomised, multicentre, open-label phase 3 trial. Lancet.

[37] Chen Q, Jain N, Ayer T, Wierda WG, Flowers CR, O’Brien SM, Keating MJ, Kantarjian HM, Chhatwal J (2017). Economic burden of chronic lymphocytic leukemia in the era of oral targeted therapies in the United States. J Clin Oncol.

[38] Kwok M, Rawstron AC, Varghese A, Evans PA, O’Connor SJ, Doughty C, Newton DJ, Moreton P, Hillmen P (2016). Minimal residual disease is an independent predictor for 10-year survival in CLL. Blood.

[39] Di Veroli GY, Fornari C, Wang D, Mollard S, Bramhall JL, Richards FM, Jodrell DI (2016). Combenefit: an interactive platform for the analysis and visualization of drug combinations. Bioinformatics.

[40] Harrington JA, Wheeler GM, Sweeting MJ, Mander AP, Jodrell DI (2013). Adaptive designs for dual-agent phase I dose-escalation studies. Nat Rev Clin Oncol.

[41] Kim ES, Herbst RS, Wistuba II, Lee JJ, Blumenschein GR, Tsao A, Stewart DJ, Hicks ME, Erasmus J, Gupta S (2011). The BATTLE trial: personalizing therapy for lung cancer. Cancer Discov.

[42] Papadimitrakopoulou V, Lee JJ, Wistuba II, Tsao AS, Fossella FV, Kalhor N, Gupta S, Byers LA, Izzo JG, Gettinger SN, Goldberg SB, Tang X, Miller VA, Skoulidis F, Gibbons DL, Shen L, Wei C, Diao L, Peng SA, Wang J, Tam AL, Coombes KR, Koo JS, Mauro DJ, Rubin EH, Heymach JV, Hong WK, Herbst RS (2016). The BATTLE-2 Study: a biomarker-integrated targeted therapy study in previously treated patients with advanced non–small-cell lung cancer. J Clin Oncol.

[43] Barker AD, Sigman CC, Kelloff GJ, Hylton NM, Berry DA, Esserman LJ (2009). I-SPY 2: an adaptive breast cancer trial design in the setting of neoadjuvant chemotherapy. Clin Pharmacol Ther.

[44] Rugo HS, Olopade OI, DeMichele A, Yau C, van ’t Veer LJ, Buxton MB, Hogarth M, Hylton NM, Paoloni M, Perlmutter J, Symmans WF, Yee D, Chien AJ, Wallace AM, Kaplan HG, Boughey JC, Haddad TC, Albain KS, Liu MC, Isaacs C, Khan QJ, Lang JE, Viscusi RK, Pusztai L, Moulder SL, Chui SY, Kemmer KA, Elias AD, Edmiston KK, Euhus DM, Haley BB, Nanda R, Northfelt DW, Tripathy D, Wood WC, Ewing C, Schwab R, Lyandres J, Davis SE, Hirst GL, Sanil A, Berry DA, Esserman LJ, I-SPY 2 Investigators (2016). Adaptive randomization of veliparib-carboplatin treatment in breast cancer. N Engl J Med.

[45] Park JW, Liu MC, Yee D, Yau C, van ‘t Veer LJ, Symmans WF, Paoloni M, Perlmutter J, Hylton NM, Hogarth M, DeMichele A, Buxton MB, Chien AJ, Wallace AM, Boughey JC, Haddad TC, Chui SY, Kemmer KA, Kaplan HG, Isaacs C, Nanda R, Tripathy D, Albain KS, Edmiston KK, Elias AD, Northfelt DW, Pusztai L, Moulder SL, Lang JE, Viscusi RK, Euhus DM, Haley BB, Khan QJ, Wood WC, Melisko M, Schwab R, Helsten T, Lyandres J, Davis SE, Hirst GL, Sanil A, Esserman LJ, Berry DA, I-SPY 2 Investigators (2016). Adaptive randomization of neratinib in early breast cancer. N Engl J Med.

[46] Kaplan R, Maughan T, Crook A, Fisher D, Wilson R, Brown L, Parmar M (2013). Evaluating many treatments and biomarkers in oncology: a new design. J Clin Oncol.

[47] Wason JMS, Abraham JE, Baird RD, Gounaris I, Vallier A-V, Brenton JD, Earl HM, Mander AP (2015). Bayesian adaptive designs for biomarker trials with biomarker discovery. Br J Cancer.

